# l-Thyroxine in an Oral Liquid or Softgel Formulation Ensures More Normal Serum Levels of Free T4 in Patients with Central Hypothyroidism

**DOI:** 10.3389/fendo.2017.00321

**Published:** 2017-11-20

**Authors:** Salvatore Benvenga, Giovanni Capodicasa, Sarah Perelli

**Affiliations:** ^1^Department of Clinical and Experimental Medicine, University of Messina, Messina, Italy; ^2^Master Program on Childhood, Adolescent and Women’s Endocrine Health, University of Messina, Messina, Italy; ^3^Interdepartmental Program on Molecular & Clinical Endocrinology, and Women’s Endocrine Health, Azienda Ospedaliera Universitaria Policlinico G. Martino, Messina, Italy

**Keywords:** central hypothyroidism, replacement therapy, undertreated hypothyroidism, oral liquid l-thyroxine, softgel l-thyroxine

## Abstract

**Context:**

l-Thyroxine (l-T4) therapy of central hypothyroidism (CH) is guided by measurements of serum free thyroxine (FT4), which should be above the midnormal range value (MNRV). In some countries, novel formulations of oral l-T4 (liquid or softgel) are available further to the classic tablets. The intestinal absorption of either novel formulation is greater than tablets in patients with primary hypothyroidism.

**Objective:**

To evaluate whether new oral formulations of l-T4 could be considered optimal in patients with CH who do not reach the FT4 target using tablet l-T4.

**Design:**

Our observation of six patients with isolated CH and serum FT4 below MNRV under stable adequate doses of tablet l-T4 (median 1.51 μg/kg bw/day), prompted us to switch them to liquid (*n* = 4) or softgel (*n* = 3) l-T4 at the same dose, and verify whether FT4 increased above MNRV. A seventh patient with FT4 above MNRV was enrolled because she wanted a “*more modern formulation*.” Postswitch FT4 was measured at least twice with the same kit as preswitch FT4.

**Results:**

In the first six patients, postswitch FT4 averaged 13.0 ± 1.6 pg/ml compared to 10.4 ± 1.8 preswitch FT4 (*P* = 0.00026), with 11/13 (85%) measurements above MNRV compared to 0/20. In the liquid or softgel l-T4 group, postswitch FT4 averaged 13.1 ± 1.6 vs. 10.6 ± 0.9 pg/ml preswitch (*P* = 0.0004) or 12.9 ± 2.1 vs. 10.3 ± 2.4 (*P* = 0.048), respectively. In the seventh patient (switched to liquid l-T4), averages were 18.3 vs. 15.2 pg/ml, and proportions 4/4 vs. 2/2.

**Conclusion:**

In CH patients, oral liquid or softgel l-T4 administered at the same doses as tablet l-T4 ensures target serum FT4 levels above MNRV that tablet l-T4 may miss. In turn, this performance suggests the more favorable pharmacokinetics profile of either novel formulation compared with the tablet formulation.

## Introduction

Based on single case reports or a few adult patients with primary hypothyroidism, we were the first to show the clinical advantage given by the new oral formulations of l-thyroxine (l-T4), *viz*. liquid or softgel capsule, compared to the classical tablet (1–3). Subsequent literature (4–10) confirmed our conclusions, which was not surprising considering the favorable pharmacokinetics profile of either liquid or softgel l-T4 (1, 3, 11, 12). Precisely, the setting in which the superiority of either novel formulations of l-T4 over tablet l-T4 was malabsorption of l-T4 caused by food, coffee, proton pump inhibitors, and calcium salts or iron salts. There are no data concerning greater performance of either novel formulation of l-T4 compared with tablet l-T4 in central hypothyroidism (CH). Therefore, the aim of this study is to evaluate whether new oral formulations of l-T4 could be considered optimal even in patients with central.

While in primary hypothyroidism, management of the l-T4 replacement therapy has to be guided by periodic measurements of serum TSH (13, 14), in CH it has to be guided by periodic measurements of serum free thyroxine (FT4) (13–15). Particularly, as recommended in two back-to-back American guidelines (13, 14), in treated CH serum FT4 should exceed the midnormal range value (MNRV) for the assay being used.

Because there are no data concerning greater performance of either novel formulation of l-T4 compared with tablet l-T4 in CH, the aim of this study is to evaluate whether new oral formulations of l-T4 could be considered optimal even in patients with CH. Here, we report our observations in patients with CH in whom serum FT4 concentrations while on tablet l-T4 replacement therapy were consistently below the mid-range level. Upon switching these patients to either novel formulation while maintaining the same daily dose of l-T4, serum FT4 passed the midrange level.

## Materials and Methods

### Patients and Methods

We targeted six ambulatory patients with CH in whom, at our observation, we realized that replacement therapy with stable doses of tablet l-T4 was associated with serum FT4 consistently below the MNRV. In addition, a seventh patient who follows the endocrine literature became aware of the availability of the oral liquid formulation at a time the softgel formulation was not yet available. Thus, she wished to switch to the liquid l-T4. In this woman with a nonfunctioning microadenoma, who was 42 years old at time of formulation switch, serum FT4 had been just marginally above the midrange level under a stable dose of 1.67 μg/kg body weight/day tablet l-T4. This woman is referred to as patient or case no. 4 throughout the article. In the remaining six patients (three women aged 48–59 and three men aged 20–74), who were under stable doses of l-T4 [1.49 ± 0.22 μg/kg bw (median 1.47)], etiologies of CH were empty sella (one woman and two men), head trauma (one woman and one man), or pituitary nonfunctional microadenoma (one woman). In all seven patients, CH was the only pituitary deficiency, so that none was under other hormone therapies. All patients used to take tablet l-T4 1 h before breakfast. Also, none of the patients had known causes of l-T4 malabsorption or was taking drugs that are known to impair the intestinal absorption of l-T4 or that influence the metabolism of T4 (16, 17). The pertinent demographics of the seven patients are summarized in Table 1. For sake of completeness of information, the seven patients belong to a series of 35 patients with isolated CH that we have seen over the years.

**Table 1:** Demographics of the seven patients with central hypothyroidism.

Patient	Age (years) at		Cause of central hypo	Body weight (kg)	Daily dose (μg/kg bw) of tablet l-T4	
	Our observation	Diagnosis of central hypo			Initial	Final^a^
1, F	48	46	Head trauma	83	1.21	1.51
2, F	57	50	Pituitary microadenoma	64	1.18	1.41
3, F	59	53	Empty sella	76	1.32	1.65
4, F	42	39	Pituitary microadenoma	60	1.50	1.67
5, M	20	16	Empty sella	55	1.18	1.29
6, M	45	44	Head trauma	82	1.22	1.22
7, M	74	69	Empty sella	70	1.43	1.83

*^a^Switch to the novel formulations of l-T4 was done at the indicated final dose*.

Absence of other pituitary deficits was based on normal levels of follicle stimulating hormone (FSH), luteinizing hormone (LH), estradiol in the two fertile age women, appropriately high levels of FSH and LH for their postmenopausal status in the two postmenopausal women, normal levels of FSH, LH, and testosterone in the three men, normal morning levels of cortisol, normal sex- and age band-specific insulin-growth factor–like 1 levels and sex-specific prolactin levels.

Upon informed written consent, patients were switched to either novel formulation they preferred. This was the oral liquid solution (Tirosint soluzione orale, by IBSA Farmaceutici Italia s.r.l., Lodi, Italy) in four patients (cases nos. 1–4) and the softgel capsule (Tirosint capsule molli, by IBSA Farmaceutici Italia s.r.l., Lodi, Italy) in the other three patients (cases nos. 5–7). The oral solution consists of predosed vials in which l-T4 is solubilized in 95% ethanol and 86% glycerol. The pearl-shaped capsule formulation contains T4 dissolved in glycerine and has gelatin as a protecting shell.

Once switched, we aimed to have at least two determinations of serum FT4. Patients were required to continue using the same laboratory they had used while on l-T4 tablets and continue to have FT4 measured on blood sample drawn in the morning after an overnight fast before taking the daily dose of l-T4. Because all patients had at least one preswitch serum TSH determination under the stable tablet l-T4 regimen, and because serum TSH levels >0.5 mU/l are considered to be suspicious for undertreated CH (15), we backed the fundamental serum FT4 assays with at least one postswitch TSH determination, again using the same laboratory.

Like other ambulatory patients who live in a vast area of north-eastern Sicily, each patient has his/her locally accessible laboratory for biochemical analyses. Once analyses are done, the patients bring results to the visit scheduled in our outpatient facility. Because several commercial FT4 assays are available, for the purpose of this article, further to absolute levels, data will be presented in a standardized fashion, namely as percent variation from the lower normal limit and percent variation from the midrange level.

### Statistics

Continuous data are reported as mean ± SD and median, with difference between means of FT4 serum levels (replacement with the new formulation vs. replacement with the classic tablet formulation) assessed by the ANOVA test. Due to the non-Gaussian distribution, difference between FT4 means of standard deviation from the midnormal range and difference between means of TSH serum levels were assessed by the Wilcoxon signed rank test.

## Results

**Figure 1:**
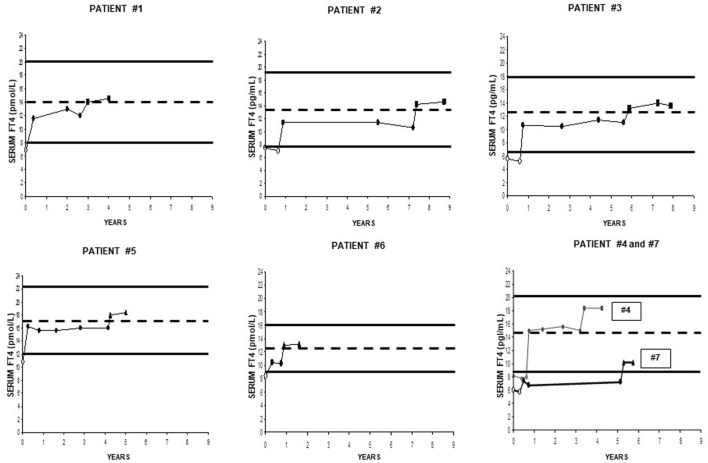
Individual serum levels of serum free thyroxine (FT4), with indicated normal reference range and (dotted line) midnormal range value. Except for two patients, serum FT4 had been measured in different laboratories for the other five patients. However, each patient used always the same laboratory, which used the same kit throughout follow-up. Empty circles (○) refer to the pretreatment period. Black circles (●) refer to the treatment period with stable doses of tablet l-T4. Black squares (■) and black triangles (▲) refer to the postswitch period, when patients were under the same daily dose of l-thyroxine but using a novel formulation (liquid or softgel capsule, respectively).

**Figure 2:**
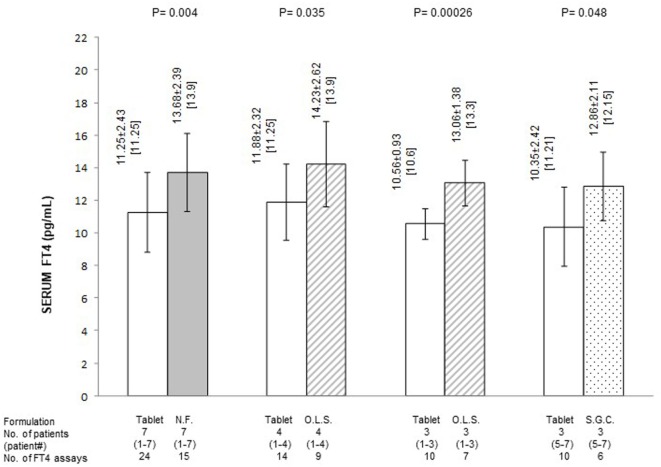
Average serum levels of free thyroxine (FT4) (pg/ml; mean ± SD and, in brackets, median) in patients stratified based on formulation of l-thyroxine. NF, novel formulation (either liquid oral solution or softgel capsule); OLS, oral liquid solution; SGC, softgel capsule. With reference to the first pair of bars, omitting patient #7 (whose postswitch serum FT4 level failed to reach the midnormal range value), the 21 preswitch and the 13 postswitch FT4 measurements averaged 11.84 ± 1.96 and 14.19 ± 2.15 pg/ml (*P* = 0.0025), with corresponding median levels of 11.5 and 14.06 pg/ml. Omitting patient #4 (whose preswitch FT4 level already passed the midnormal range value), the 20 preswitch and the 13 postswitch FT4 measurements averaged 10.45 ± 1.79 (median = 10.6) and 12.97 ± 1.58 pg/ml (median 13.9) (*P* = 0.0004).

**Figure 3:**
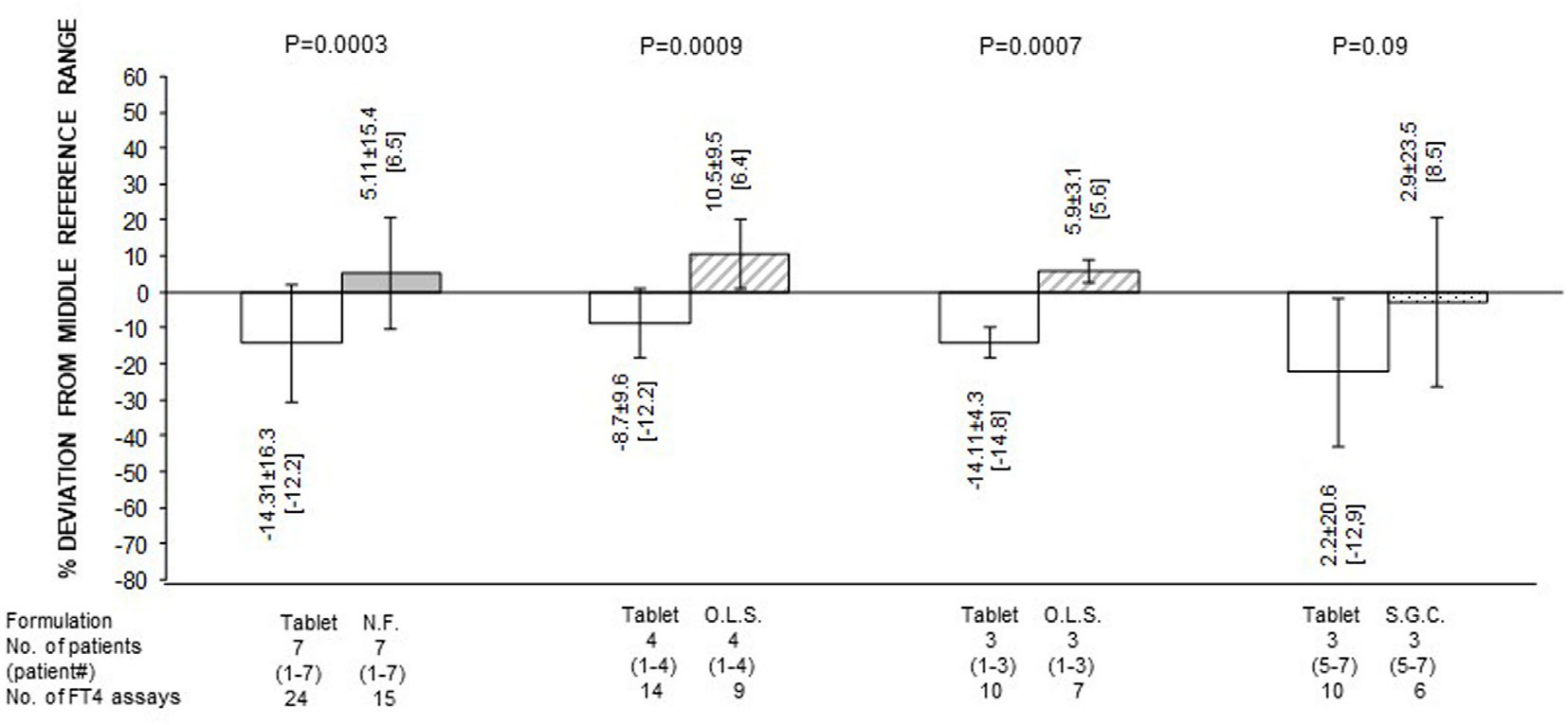
Serum free thyroxine (FT4) as percent deviation from the midnormal range value (mean ± SD and, in brackets, median) in patients stratified based on formulation of l-thyroxine. NF, novel formulation (either liquid oral solution or softgel capsule); OLS, oral liquid solution; SGC, softgel capsule. With reference to the first pair of bars, omitting patient #7 (whose postswitch serum FT4 level failed to reach the midnormal range value), the 21 preswitch and the 13 postswitch FT4 measurements, as percent deviation from the midnormal range value, averaged −9.1 ± 8.5 and 10.25 ± 7.9 pg/ml (*P* = 1 × 10^−5^), with corresponding median levels of −8.8 and 7.4 pg/ml. Omitting patient #4 (whose preswitch FT4 level already passed the midnormal range value), the 20 preswitch and the 13 postswitch FT4 measurements, as percent deviation from the midnormal range value, averaged −18.14 ± 15.0 and 1.82 ± 13.7 pg/ml (*P* = 0.0004), with corresponding median levels of −14.8 and 6.4 pg/ml.

**Figure 4:**
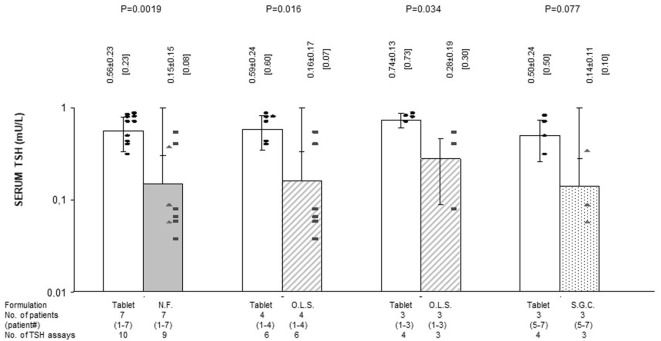
Average serum levels of TSH (mU/l; mean ± SD and, in brackets, median) in patients stratified based on formulation of l-thyroxine. NF, novel formulation (either liquid oral solution or softgel capsule); OLS, oral liquid solution; SGC, softgel capsule. For the symbols, see Figure 1. Black circles (●) refer to the treatment period with stable doses of tablet l-T4. Black squares (■) and black triangles (▲) refer to the postswitch period, when patients were under the same daily dose of l-thyroxine but using a novel formulation (liquid or softgel capsule, respectively). With reference to the first pair of bars, omitting patient #7 (whose postswitch serum FT4 level failed to reach the midnormal range value), the nine preswitch and the eight postswitch TSH measurements averaged 0.58 ± 0.26 and 0.17 ± 0.15 mU/l (*P* = 0.0039), with corresponding median levels of 0.60 and 0.09 mU/l. Omitting patient #4 (whose preswitch FT4 level already passed the midnormal range value), the eight preswitch and the six postswitch TSH measurements averaged 0.62 ± 0.22 and 0.21 ± 0.22 mU/l (*P* = 0.012), with corresponding median values of 0.65 and 0.65 mU/l.

Individual data are illustrated in Figure 1, and summarized in Figures 2–4. All patients but two (nos. 4 and 7) had serum FT4 under replacement with tablet l-T4 that was always below the MNRV (Figure 1). Patient no. 4 wished to be switched to the liquid l-T4 because desiring to be treated with “*a more modern formulation*” (see Patients and Methods). Patient no. 7 had FT4 even below the lower normal limit, in face of the relatively high daily dose of tablet l-T4 (1.83 μg/kg bw). After a thorough diagnostic work-up (16), the reason for such apparent malabsorption of l-T4 in this patient remains unclear, similarly to approximately 15% of patients with primary hypothyroidism who come to observation for undertreated, refractory hypothyroidism (16). Upon maintaining both the same daily dose of l-T4, but using either novel formulation of l-T4 and the same FT4 assay, serum FT4 levels were invariably greater than under the tablet l-T4 regimen in all seven patients (Figure 1). No patient complained of palpitations.

Quantification of the changes in FT4 in terms of both absolute levels and percent deviation from the MNRV is presented in Figures 2 and 3, respectively. In these figures, patients are also stratified based on the novel formulation they were switched to. Data for patients who were switched to liquid l-T4 (cases nos. 1–4) are also presented by omitting case no. 4, as this patient already had serum FT4 above the MNRV while on tablet l-T4. In the comparison of serum FT4 levels between the preswitch period and the postswitch period, the corresponding means ± SD were always statistically significant. Only, in the comparison of the percent deviation of FT4 from the midrange normal value between the tablet and the softgel l-T4 (Figure 3), the difference was borderline significant (*P* = 0.09), due to the large standard deviation conferred by patient no. 7.

In summary, regardless of the novel formulation used, serum FT4 levels are approximately 20–25% greater compared to the tablet formulation (Figure 1). Excluding case no. 4 and the outlier case no. 7, the midrange normal value was always reached under either novel formulation (Figures 1 and 3).

Preswitch and postswitch TSH levels are illustrated in Figure 4 and are the mirror image of FT4 levels shown in Figures 2 and 3.

## Discussion

According to authors experts in CH, the l-T4 dose should be “*about 1.4–1.7* μ*g/kg bw*” (15). Based on the 2012 guidelines for the management of hypothyroidism in adults (13), in CH estimates of dosage based on 1.6 μg/kg bw l-T4 daily and assessment of FT4 should guide therapy. The same 2012 guidelines recommend that “*In patients with central hypothyroidism, assessment of serum FT4 should guide therapy and targeted to exceed the midnormal range value for the assay being used*” (13). Based on the 2014 guidelines (14), “*The dilemma with respect to treating secondary hypothyroidism is that it cannot be determined what is the ‘normal’ FT4 for the individual patient*.”

The same guidelines (14) state that “*In patients with secondary hypothyroidism, the primary biochemical treatment goal should be to maintain the serum free thyroxine values in the upper half of the reference range. However, the serum free thyroxine target level may be reduced in older patients or patients with comorbidities, who may be at higher risk of complications of thyroid hormone excess*.” However, these guidelines (14) admit that “*Other recommendations in the literature include keeping FT4 concentrations within the reference range (ref.), keeping FT4 levels in the middle of the laboratory reference range (refs), and keeping the FT4 levels in the same range as the FT4 levels seen in patients being treated for primary hypothyroidism (ref)*.”

In the present article on a group of patients with CH, we show an evident superiority of either novel formulation of l-T4 compared to the classical l-T4 tablets in achieving more normal circulating levels of FT4. This evidence received another support by the measurement of serum TSH. Nevertheless, instead of switching to novel formulations, one can continue to stepwise increase the daily dose of tablet l-T4 until reaching target levels. However, this approach prolongs the time of undertreatment or incomplete treatment of CH and requires more assays of serum FT4.

In addition to intra-subject adherence to the same assay of FT4 in the same laboratory and having at least two measurements of serum FT4 both preswitch and postswitch, one other strength of our work is to have evaluated only patients with CH under l-T4 monotherapy. Indeed, concomitant treatment of additional pituitary deficiencies with other hormones (glucocorticoids, sex hormones, and recombinant GH) affects T4 metabolism and may cause an increased requirement of l-T4 daily dose. A further strength is to have supported changes of serum FT4 with changes, in the opposite direction, of serum TSH. Considering the rarity of secondary hypothyroidism compared to primary hypothyroidism, and even more so the rarity of isolated CH, the size of our series (*n* = 7) is only apparently small.

One side-consideration we wish to make is that, similarly to refractory primary hypothyroidism (16), refractory CH (as defined by serum FT4 remaining below the lower normal limit of the laboratory assay being used) exists too, and with a similar frequency [approximately 20% compared with 14% (1 out of 7) in our series of patients].

## Conclusion

In almost 90% of patients with isolated CH, either liquid l-T4 or softgel capsule l-T4 permits to reach target levels of serum FT4.

## Ethics Statement

This study was carried out with written informed consent from all subjects. All subjects gave written informed consent in accordance with the declaration of Helsinki. Because patients recruited in the study underwent the regular work-up of hypothyroid subjects and were not submitted to life-risk procedures, the study protocol was not submitted to the Ethical Committed of the authors’ Institutions in accordance with the institutional and national requirements.

## Author Contributions

All the authors contributed equally to recruited patients and wrote the present work. SB collected data and performed statistical analysis.

## Conflict of Interest Statement

IBSA Institut Biochimique SA (Lugano, Switzerland) and IBSA Farmaceutici Italia s.r.l. furnished the principal investigator (SB) with novel formulations to conduct studies cited in the reference list. However, IBSA had no role in any phase of the writing of the above and this manuscript. Furthermore, SB was an invited speaker at symposia organized by IBSA.
